# Comparative genome analysis of *Phyllosticta citricarpa* and *Phyllosticta capitalensis*, two fungi species that share the same host

**DOI:** 10.1186/s12864-019-5911-y

**Published:** 2019-07-05

**Authors:** Carolina Munari Rodrigues, Marco Aurélio Takita, Nicholas Vinicius Silva, Marcelo Ribeiro-Alves, Marcos Antonio Machado

**Affiliations:** 1 Laboratório de Biotecnologia, Centro APTA Citros Sylvio Moreira, CP4, Cordeirópolis, SP 13490-970 Brazil; 2 Laboratório de Genômica e BioEnergia, Universidade Estadual de Campinas, Campinas, SP 13083-970 Brazil; 3 Laboratório de Pesquisa Clínica em DST/AIDS, Instituto Nacional de Infectologia Evandro Chagas, Fundação Oswaldo Cruz, Rio de Janeiro, RJ 21040900 Brazil

**Keywords:** Pathogenic species, Endophytic species, Citrus black spot, Functional enrichment analysis, Phylogeny, Gene family expansion

## Abstract

**Background:**

Citrus are among the most important crops in the world. However, there are many diseases that affect Citrus caused by different pathogens. Citrus also hosts many symbiotic microorganisms in a relationship that may be advantageous for both organisms. The fungi *Phyllosticta citricarpa*, responsible for citrus black spot, and *Phyllosticta capitalensis*, an endophytic species, are examples of closely related species with different behavior in citrus. Both species are always biologically associated and are morphologically very similar, and comparing their genomes could help understanding the different lifestyles. In this study, a comparison was carried to identify genetic differences that could help us to understand the biology of *P. citricarpa* and *P. capitalensis*.

**Results:**

Drafts genomes were assembled with sizes close to 33 Mb for both fungi, carrying 15,206 and 14,797 coding sequences for *P. citricarpa* and *P. capitalensis*, respectively. Even though the functional categories of these coding sequences is similar, enrichment analysis showed that the pathogenic species presents growth and development genes that may be necessary for the pathogenicity of *P. citricarpa*. On the other hand, family expansion analyses showed the plasticity of the genome of these species. Particular families are expanded in the genome of an ancestor of *P. capitalensis* and a recent expansion can also be detected among this species. Additionally, evolution could be driven by environmental cues in *P. citricarpa*.

**Conclusions:**

This work demonstrated genomic differences between *P. citricarpa* and *P. capitalensis*. Although the idea that these differences could explain the different lifestyles of these fungi, we were not able to confirm this hypothesis. Genome evolution seems to be of real importance among the *Phyllosticta* isolates and it is leading to different biological characteristics of these species.

**Electronic supplementary material:**

The online version of this article (10.1186/s12864-019-5911-y) contains supplementary material, which is available to authorized users.

## Background

*Phyllosticta citricarpa* McAlpine (Synonym: *Guignardia citricarpa* Kiely) is the fungus responsible for causing citrus black spot (CBS), which is one of the most important diseases affecting the citrus industry worldwide [1, 2]. This disease was first described in Australia, where it caused considerable losses for sweet orange growers [3]. Since then, CBS has seriously affected citrus crops in countries in Africa, Asia, South America, and North America, especially in Argentina, United States and Brazil [1, 2, 4–6].

CBS affects almost all commercial varieties of citrus with the main symptom associated with this disease being the development of hard spot lesions in the fruit peel [7]. Advanced stages of the disease lead to maturation and early fall of the fruit [5]. In addition, the affected fruits have their appearance depreciated, making them unsuitable for the fresh fruit market, and therefore the costs associated with chemical control of the disease are significant [1, 7–9].

Most of the species in the genus *Phyllosticta* are plant pathogens of a wide range of hosts [10, 11] and although *P. citricarpa* is pathogenic, other endophytic and saprophytic species have also been reported for citrus [12–15]. *P. capitalensis* is among the endophytic species that lives within citrus and other hosts with a wide geographic distribution [10, 16, 17]. *P. capitalensis* is commonly found to be associated with lesions in plants; from an economic point of view, this association can be very negative since *P. capitalensis* is normally confused with the pathogen that is actually responsible for causing the disease. This is an even worse problem if the actual pathogen is a quarantine organism like *P. citricarpa* that has phytosanitary restrictions, being classified as quarantine A1 in the European Union and A2 in the United States [4, 18]. The similarity between *P. citricarpa* and *P. capitalensis* is so close that CBS was erroneously reported in New Zealand [2, 19], with the endophytic species being identified as the species responsible for causing the disease [20]. Due to the high similarity between these species, identifications can only be made at the molecular level [21, 22].

In recent years, the number of works using comparative genomic analysis to understand the genetic basis of the lifestyle of pathogenic and endophytic or symbiont organisms has grown considerably [23–26]. In this study, the genome of the citrus pathogenic species *P. citricarpa* was compared to the genome of *P. capitalensis*, the endophytic species, in order to identify genetic differences that could help understand their different lifestyles.

## Results and discussion

### Overview of genome sequencing and categorization

The Illumina sequencing of *P. citricarpa* and *P. capitalensis* genomes generated a total of 179,880,616 and 148,831,020 paired-end reads, respectively, with more than 90% showing Phred quality > 20 (Q20) for both species. The CG content was slightly lower for *P. citricarpa* (48.72) compared to *P. capitalensis* (51.43).

A de novo assembly was done for both genomes, generating drafts of 19,143 contigs in 32.6 Mb for *P. citricarpa* and N50 of 3049. The assembly of *P. capitalensis* reads resulted in 11,080 contigs and 33.2 Mb with a N50 of 4925. A Genome Shotgun project has been deposited at DDBJ/EMBL/GenBank under the BioProject number PRJNA486917.

The contigs of both fungi were used for the prediction of coding sequences using *Botrytis cinerea*, an ascomycete necrotrophic plant pathogen with a broad host range [27] as a reference in Augustus [28]. For *P. citricarpa,* 15,206 proteins were identified, while for *P. capitalensis,* the total was 14,797. These proteins were annotated using blastp [29] to find similarities with subjects in the Protein database of the National Center for Biotechnology Information (https://www.ncbi.nlm.nih.gov/protein/) (Additional files 1 and 2). Further characterization of the putative proteins was performed in Blast2GO [30], and when they were distributed in different categories, i.e.*,* cellular component, molecular function, and biological process (level 2).

**Fig. 1:**
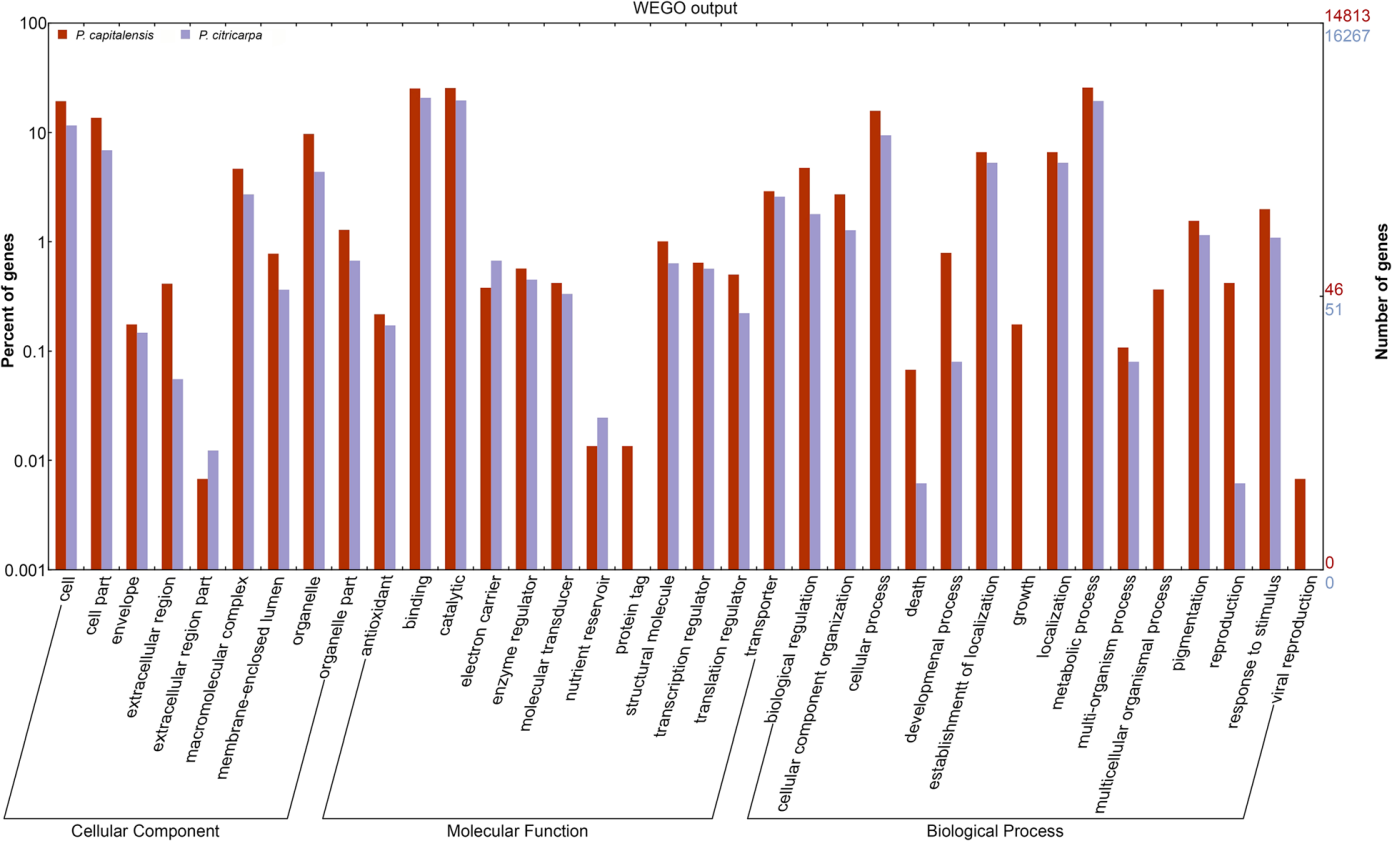
Categorization of all genes of *P. citricarpa* and *P. capitalensis*. The genes were automatically classified based on GO (Gene Ontology) and were distributed according to their function performed in fungus cells. The genes were distributed into three categories: cellular component, molecular function and biological process annotation. *P. capitalensis* (red bars) and *P. citricarpa* (blue bars)

Even though the distribution of the genes of *P. citricarpa* and *P. capitalensis* showed the same pattern for most of the subcategories, with a few additional genes in the latter compared to the former, there were some differences that could be representative for understanding the behavior of both organisms (Fig. 1). In the molecular function category, the binding and catalytic subcategories presented the highest percentage of genes in both genomes while the protein tag subcategory was present only in *P. capitalensis* (Fig. 1).

For biological processes, the most representative sub categories were metabolic processes, cellular processes, establishment of localization, and localization. The growth, multicellular organismal processes and viral reproduction subcategories were unique for the endophytic fungus and in addition, it also showed a higher percentage of genes in the death and reproduction subcategories (Fig. 1).

BUSCO analysis confirms that the genomes are not complete with 502 and 765 proteins missing for *P. capitalensis* and *P. citricarpa*, respectively (Additional file 3) in a total of 3156 proteins. Other genomes of *Phyllosticta* spp. are much more complete and therefore were used for validating the results obtained with our genomes.

### Phylogenetic analysis of *Phyllosticta* species

**Fig. 2:**
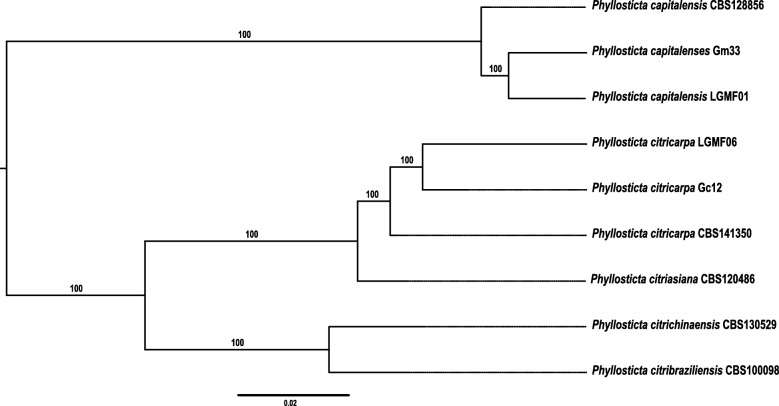
Maximum likelihood tree generated from genomic sequence analysis of the *Phyllosticta* species. The maximum likelihood phylogenetic analysis was performed using 3.185 single copy-ortholog genes with support values obtained for 1000 bootstrap replicates using the JTT + F + R4 model. Bootstrap support values for maximum likelihood (ML) are shown above the branches. The root of ML tree was inferred the midpoint rooting method

To verify the evolutionary relationship of different *Phyllosticta* species, we used the two genomes sequenced in this work together with seven other genomes available in public databases (*P. capitalensis* strains: CBS128856, Gm33, and LGMF01; *P. citricarpa* strains: CBS141350, Gc12, and LGMF06, *P. citribraziliensis* strain CBS100098, and *P. citrichinaensis* strain CBS130529). The phylogenetic tree obtained using the 3185 single copy-ortholog genes (Fig. 2) confirms that *P. citricarpa* and *P. citriasiana* are very closely related and that *P. capitalensis* is distant from this group, confirming the result obtained with ITS, LSU, TEF1, ACT and GPDH sequences alignment [10, 31]. *P. capitalensis* and *P. citribraziliensis* are endophytes in *Citrus* while *P. citricarpa* and *P. citriasiana* are pathogenic. *P. citrichinaensis*, on the other hand, causes minor disease symptoms [32] and therefore, pathogenicity in *Citrus* may be related to the phylogeny of *Phyllosticta* species at least in part since the two pathogenic species cluster together.

### Major functional categories enriched in *P. citricarpa* compared with *P. capitalensis*

**Fig. 3:**
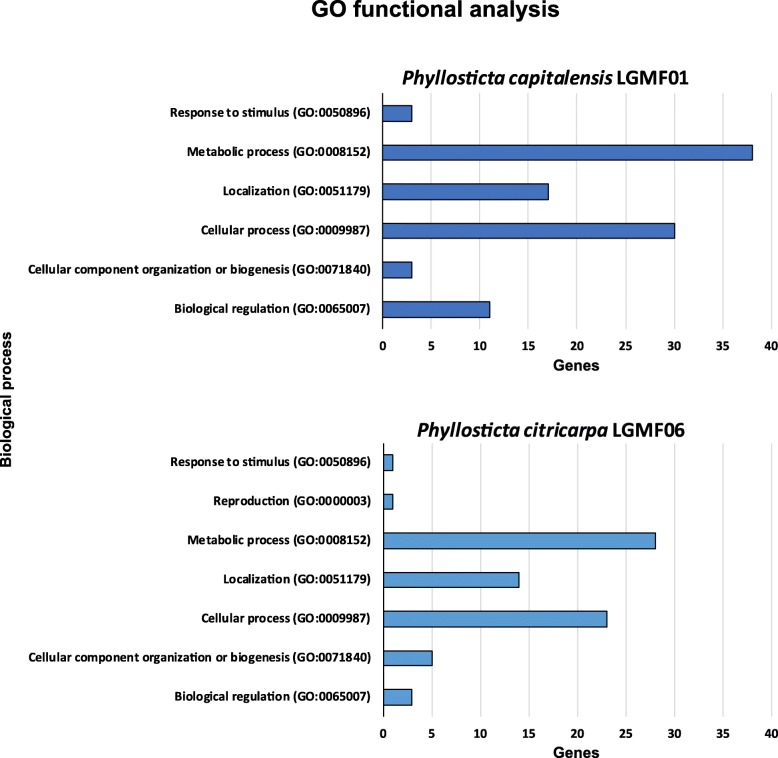
PHANTHER GO-Slim biological process subcategories enriched in *P. capitalensis* LGMF6 and *P. citricarpa* LGMF01. List of subcategories obtained for *Phyllosticta* species in enrichment analysis using the gene set enrichment analysis. Each category is associated with the number of genes from each fungi in comparison to the total number of genes of the reference organism (*Saccharomyces cerevisiae*) with the same GO term. Note that the same gene may have more than one associated ontology

To better evaluate the differences at the genetic level between one pathogenic and one endophytic species, the sequences from *P. citricarpa* and *P. capitalensis* were used in a functional enrichment analysis to verify potential molecular mechanisms associated with their interaction with citrus (Fig. 3).

It was observed that enrichment analysis resulted in a difference between *P. citricarpa* and *P. capitalensis*. For the Biological Process category, *P. citricarpa* showed a higher number of enriched subcategories, GO:0044848, GO:0051704, GO:0000003, and GO:0032502, which are all related with growth and development. This result may indicate that *P. citricarpa* presents a more complex living compared to *P. capitalensis*, which could result from the distant relationship in evolution. However, even though it is an appealing hypothesis for testing, it would be necessary to evaluate other sequenced genomes that show closer phylogenetic relationship to *P. capitalensis*. Other works reporting the colonization of *P. capitalensis* and *P. citricarpa* in *Citrus* are also missing, in particular those of global gene expression.

### Gene families expansion in *Phyllosticta*

**Fig. 4:**
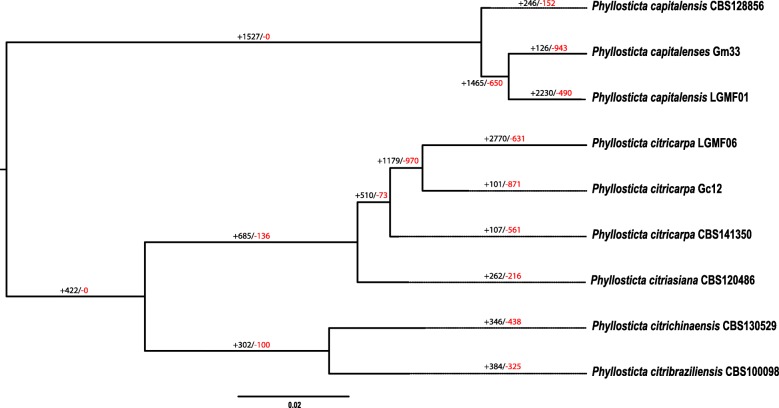
Phylogenetic inference and gains/losses of gene for *Phyllosticta* species. Number of gene gains and losses are shown on tree branches and indicated with + and −, respectively

We also did an analysis of gene families expansion in the genomes of *Phyllosticta* species. This analysis was done based on the phylogeny with the five different species of *Phyllosticta* used in this work (Fig. 4). The analysis of gene family expansion shows a high number of families being expanded from ancestral 5 in relation to 17, in the *P. capitalensis* clade (Additional file 4). This seems to be much more related to the species evolution than the lifestyle of the organisms since *P. citribraziliensis*, a *Citrus* endophyte, is not in the same clade of *P. capitalensis*. In addition, a family of HET-domain containing proteins is expanded in all the *P. capitalensis* analyzed, being a more recent event in evolution of the species. HET proteins were identified as important for vegetative incompatibility, in the formation of heterokaryon [33]. These proteins induce cell death avoiding the formation of heterokaryon [34]. Therefore this genomic data make us believe that *P. capitalensis* may be more effective in avoiding heterokaryon formation than the other *Phyllosticta* species.

In the other clade, fewer families were expanded and besides, most of them encodes uncharacterized proteins (Fig. 4). However it is worth mentioning the expansion of a family of putative chitin recognition proteins that happens just in the *P. citricarpa* genome reported in this work (LGMF06). LysM domain proteins bind chitin and are important for fungus defense against the host response [35]. Because the expansion occurs just in the isolate sequenced in our lab, we suggest that the environment may have an importance in modulating the genome plasticity in *P. citricarpa*, as known for other filamentous fungi [36].

### Comparative genomics

The phylogenetic analysis with all the genes confirmed that indeed *P. citricarpa* and *P. capitalensis* are distant species as previously reported. Therefore, the changes observed in their genomes may result from the evolution of the species, including gain and loss of genes. Even distant, a comparative genomics analysis is appealing because of the different lifestyle and it was carried out to identify genes exclusively found in the genomes of *P. citricarpa* LGMF06 and *P. capitalensis* LGMF01. A reciprocal blastp analysis was carried out using the protein sequences encoded in both genomes. The exclusive proteins were considered those that either had a positive hit with an E-value above 1 e^− 30^ or resulted in a “no hit”. These proteins were reciprocally re-evaluated in blastp using the proteomes from *P. citricarpa* CBS141350 and *P. capitalensis* CBS128856 to make sure that the differences were not a problem resulting from the incompleteness of the genomes (Table 1; Additional files 5 and 6). These genes were further evaluated in order to better understand the pathogenicity of *P. citricarpa*.

**Table 1:** Number of exclusive proteins for *P. citricarpa* and *P. capitalensis* after analyses by blastp

Total proteins of the genome		Unique proteins	
*P. citricarpa*	*P. capitalensis*	*P. citricarpa*	*P. capitalensis*
15206	14797	2896	4164

### Genes related to pathogenicity

To identify putative genes involved in pathogenicity, we analyzed the *P. citricarpa* and *P. capitalensis*-exclusive genes for similarity with subjects in the pathogen-host interaction gene database (PHI-base).

Of the 2896 gene sequences of *P. citricarpa* (Additional file 5), 123 were identified as having similarity to putative PHI proteins (Additional file 7). For *P. capitalensis* this total was 210 out of 4164 exclusive genes (Additional files 6 and 8).

**Fig. 5:**
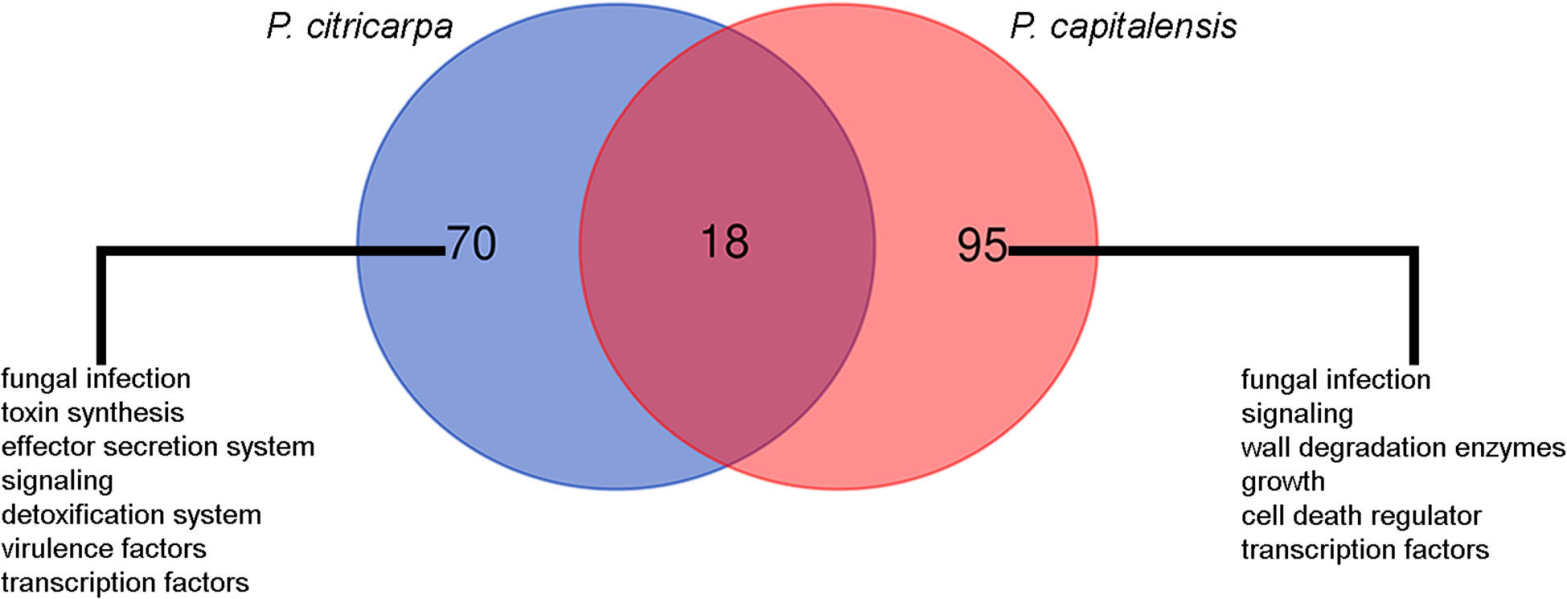
Pathogenicity gene orthologs in *P. citricarpa* and *P. capitalensis* in comparison to other fungal species using PHI-base data**.** Distribution of putative *P. citricarpa*, *P. capitalensis* and common genes involved in pathogenicity

Among the 123 genes of *P. citricarpa* and 210 of *P. capitalensis*, 18 genes are common to both fungi (Fig. 5; Additional file 9). Therefore, 70 genes were unique in the pathogenic species, while 95 were exclusive to the citrus endophytic species (Fig. 5; Additional file 9).

Among the PHI exclusive genes of *P. citricarpa*, genes involved in fungus infection, toxin synthesis, effector protein secretion system (important for the pathogenicity of pathogenic organisms), signaling, detoxification systems, virulence factors and transcription factors were identified (Additional file 5). Because the role of these genes in pathogenicity and virulence is already known for other interactions, we assume that they are important in the development of CBS in citrus [37].

*P. capitalensis* also presents genes involved in fungal infection, cell wall degradation, growth, cell death regulation and signaling (Additional file 6). Although this fungus is an endophyte in citrus and a weak plant pathogen with a worldwide distribution presently known from 70 plant families [17], it is the causal agent of the leaf spot in the orchid *Bifrenaria harrisoniae* [14], which explains the presence of pathogenicity genes in the genome of this fungus.

The molecular components of *Phyllosticta* spp. pathogenicity in citrus are poorly known. In fact to our knowledge there is one report of identification of putative proteins involved in pathogenicity of *P. citricarpa* in citrus [38], in which the authors suggest a possible major role of pectinases for this organism. Therefore, this work opens new perspectives in understanding the pathogenicity of these *Phyllosticta* species.

### Analysis of Carbohydrate-Active Enzymes (CAZymes) in *P. citricarpa* and *P. capitalensis.*

The function of CAZymes is breakdown, biosynthesis or modification of glycoconjugates, oligo- and polysaccharides. These enzymes are produced by phytopathogens and play a central role in the breakdown and synthesis of plant cell walls during host-pathogen interactions [39]. Due to the importance of CAZymes in fungal pathogenicity, we examined the presence of these enzymes in both fungi, comparing and evaluating the exclusive genes of each species.

**Fig. 6:**
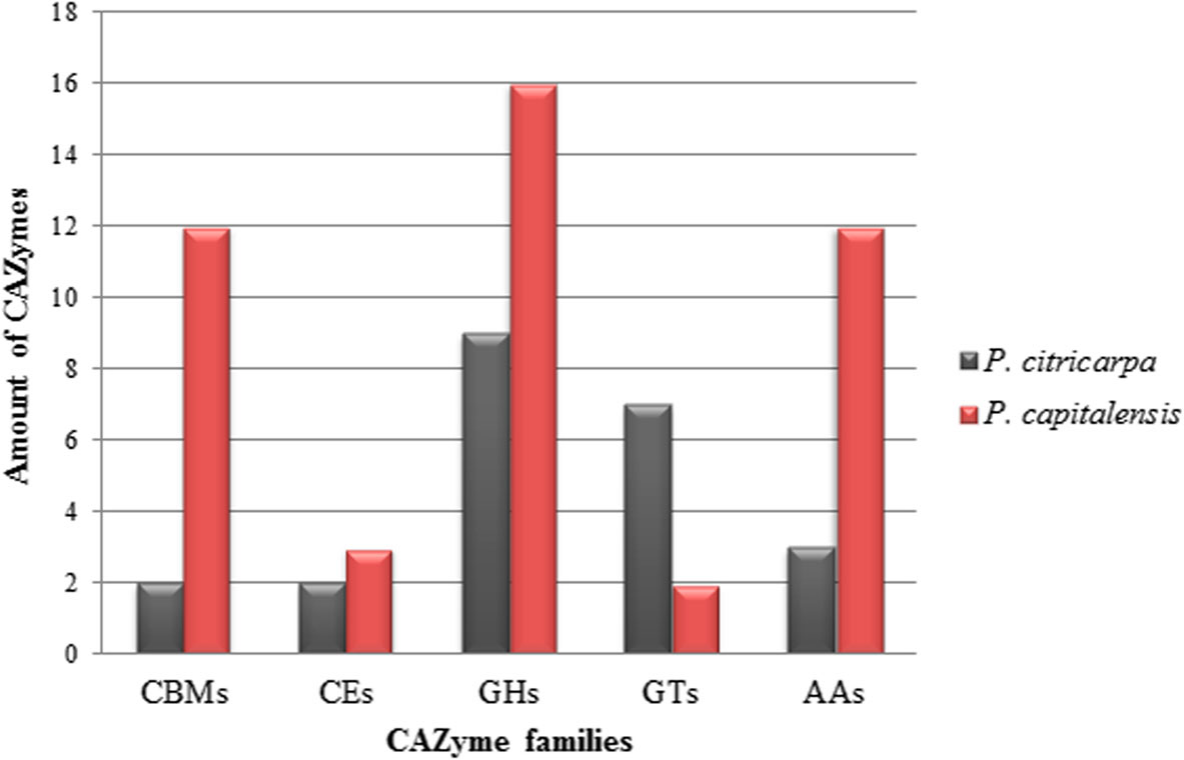
Comparative analysis of CAZymes in *P. citricarpa* and *P. capitalensis*. Number of CAZymes in *P. citricarpa* compared to *P. capitalensis* and their distributions among different families. CBM, carbohydrate-binding module; CE, carbohydrate esterases; GHs, glycoside hydrolases; GTs, glycosyl transferases; AAs, auxiliary activities

The search for CAZymes revealed 23 in *P. citricarpa*, which were distributed across CBMs (carbohydrate-binding modules), CEs (carbohydrate esterases), GHs (glycoside hydrolases), GTs (glycosyl transferases) and AAs (auxiliary activities) families (Fig. 6, Table 2). It is also noted there was diversity within each family (and subfamily), mainly GTs and GHs (Table 2).

**Table 2:** Overview of CAZyme and number of genes in each CAZyme category. Color tones differentiate families from enzymes

ID *P. citricarpa* (aa)	CAZymes Family
p.citric. LGMF06_1852	AA10
p.citric. LGMF06_14427	AA5
p.citric. LGMF06_1084	AA9
p.citric. LGMF06_1084	CBM1
p.citric. LGMF06_14427	CBM18
p.citric. LGMF06_14427	CE4
p.citric. LGMF06_13085	CE9
p.citric. LGMF06_2729	GH16
p.citric. LGMF06_0566	GH17
p.citric. LGMF06_14427	GH19
p.citric. LGMF06_12138	GH30_7
p.citric. LGMF06_14013	GH31
p.citric. LGMF06_12570	GH37
p.citric. LGMF06_1320	GH4
p.citric. LGMF06_13007	GH4
p.citric. LGMF06_6360	GH4
p.citric. LGMF06_10292	GT2
p.citric. LGMF06_1320	GT2
p.citric. LGMF06_13007	GT2
p.citric. LGMF06_15124	GT2
p.citric. LGMF06_4508	GT2
p.citric. LGMF06_6360	GT2
p.citric. LGMF06_3350	GT34
ID *P. capitalensis* (aa)	CAZymes Family
p.capi. LGMF01_10165	AA5_1
p.capi. LGMF01_12968	AA1_3
p.capi. LGMF01_0108	AA11
p.capi. LGMF01_10555	AA3_2
p.capi. LGMF01_10630	AA3_2
p.capi. LGMF01_10952	AA3_2
p.capi. LGMF01_11995	AA3_2
p.capi. LGMF01_4648	AA3_2
p.capi. LGMF01_7130	AA3_2
p.capi. LGMF01_9145	AA3_2
p.capi. LGMF01_9405	AA3_2
p.capi. LGMF01_13226	AA7
p.capi. LGMF01_12450	CBM1
p.capi. LGMF01_6291	CBM1
p.capi. LGMF01_8576	CBM1
p.capi. LGMF01_13173	CBM13
p.capi. LGMF01_9957	CBM13
p.capi. LGMF01_9957	CBM20
p.capi. LGMF01_13173	CBM32
p.capi. LGMF01_7886	CBM48
p.capi. LGMF01_10165	CBM50
p.capi. LGMF01_0072	CBM50
p.capi. LGMF01_13173	CBM57
p.capi. LGMF01_3297	CBM63
p.capi. LGMF01_14357	CE4
p.capi. LGMF01_1089	CE5
p.capi. LGMF01_9188	CE6
p.capi. LGMF01_6291	GH10
p.capi. LGMF01_13896	GH16
p.capi. LGMF01_8576	GH16
p.capi. LGMF01_11414	GH17
p.capi. LGMF01_6680	GH18
p.capi. LGMF01_9957	GH18
p.capi. LGMF01_9957	GH23
p.capi. LGMF01_12450	GH3
p.capi. LGMF01_13207	GH3
p.capi. LGMF01_9122	GH3
p.capi. LGMF01_9188	GH43_35
p.capi. LGMF01_3143	GH5_7
p.capi. LGMF01_10165	GH55
p.capi. LGMF01_10165	GH71
p.capi. LGMF01_3563	GH76
p.capi. LGMF01_5203	GT1
p.capi. LGMF01_8703	GT2

The diversity within the families in the pathogenic species was also higher in relation to endophytic species (Fig. 6, Table 2). *P. capitalensis* presents 44 genes coding for CAZymes distributed among CBM, CE, GH, GT and AA families, the latter being the only one to present more genes in this species (Fig. 6, Table 2).

We analyzed which of these CAZymes may be secreted by *P. citricarpa* and *P. capitalensis*. Of the 128 enzymes of the pathogenic species, 4 are possibly secreted: 1 of the GT family (34), and 3 of the GH family (16, 17, and 37) (Additional file 10). The endophytic species presented 1 GBM (63) secreted, 3 enzymes of the CE family (4, 5, and 6) and 5 proteins of the family GH (16, 18, 3, 43, and 55), totaling 9 CAZymes secreted (Additional file 10). The results show that *P. capitalensis* presents a greater amount of these enzymes in relation to *P. citricarpa*, besides presenting a higher diversity of representatives of the different CAZymes families. Since these two species present high similarity and identification can only be made at the molecular level [21, 22], the divergency between the two species regarding CAZymes may be an important distinguishing characteristic, allowing the development of new markers for differencing the two species, like antibodies for instance.

Most of the enzymes secreted by *P. citricarpa* are from the GH family. These are involved in hydrolysis of glycosidic bonds between or within carbohydrate molecules [39].

The fact that CAZymes are responsible for the breakdown of cell wall components suggests they are strictly related to the successful infection process of the fungus. The number of CAZymes is higher in pathogenic fungi [26]. These results are in agreement with those found by [38] where it was verified that *P. citricarpa* produces more endoglucanases in relation to *P. capitalensis*, in addition to producing more amylases and pectinases. The authors concluded that these differences could be associated with differences in pathogenicity for citrus plants. Similar to classical necrotrophic fungi, such as *Botrytis cinerea* and *Sclerotinia sclerotiorum*, the *P. citricarpa* genome is predicted to encode a large number of CAZymes involved in plant cell wall degradation [40, 41]. However, because *P. capitalensis* has more CAZymes in its genome compared to *P. citricarpa*, we could not do any association of the number of these enzymes with the lifestyle of the fungi. But the possibly secreted CAZymes of *P. citricarpa* is different from the ones from *P. capitalensis*, which could have an influence in the pathogenicity of the fungus.

All of our analyses could not give enough support to state that the different lifestyle could be explained by the genome differences observed between *P. citricarpa* and *P. capitalensis*. Indeed there is a debate on whether endophytes are pathogenic or not, or may turn pathogenic under certain conditions [42].

## Conclusion

There are many different genomes sequenced for *Phyllosticta* species. In this work we presented the partial genomes of two isolates, one from *P. citricarpa* and another one from *P. capitalensis*, which present different lifestyles and are also distantly related in evolution*.* Different features were identified in each genome and could be used for understanding a little more about the biology on the different fungi. Nevertheless some of these features seem to be related to the phylogeny of the group and it would be very interesting to have more genomes available from other closely related species in special to *P. capitalensis* to confirm these findings. However, our analyses showed interesting trends like the HET-domain containing proteins expansion in all the *P. capitalensis* strains or the expansion of a family of putative chitin recognition proteins in the *P. citricarpa* genome reported in this work (LGMF06). These findings seem to be related to the evolution of the species detected through the genome analyses but drivers are acting in the genome of the different species that could leads to the formation of new species in the future.

## Methods

### Fungal isolates, culture condition and DNA isolation

The fungi isolates used for genome sequencing are from the biological bank of the Laboratory of Microorganisms (LabGeM) at the Federal University of Paraná, Curitiba, Paraná, Brazil. The pathogenic species *P. citricarpa* (LGMF06) was isolated from CBS lesions in fruits from a plant grown in the State of São Paulo, Brazil, and tested for pathogenicity according to [43]. The endophytic species *P. capitalensis* (LGMF01) was isolated from asymptomatic fruits from a plant also grown in the State of São Paulo, Brazil. These fungi were grown in liquid Citrus Medium Fabris-Nishimura (CFN) [44] and incubated under agitation (65 rpm) at 25 °C for 7 days in the dark. The mycelium was removed from the medium, washed with distilled water, macerated under liquid nitrogen and stored at − 80 °C for later use in the DNA extraction procedure [12].

### Genome sequencing, assembly and annotation

A total of 10 μg of DNA from each fungus (*P. citricarpa* and *P. capitalensis*) was sent to Macrogen Inc. (South Korea) for sequencing using the HiSeq 2000 platform (Illumina Inc.). All procedures were performed according to Illumina’s protocols. Paired-end fragments were generated with 101 base pairs (bp). One lane was used per library. The results were sent in fastq format. De novo assembly was carried out using CLC Genomics Workbench software (https://www.qiagenbioinformatics.com/products/clc-genomics-workbench/) with default parameters.

The prediction of open read frames (ORFs) was conducted using Augustus software v3.2 [32] with *Botrytis cinerea* (*Botryotinia fuckeliana*) as the model species. The annotation and categorization of ORFs were automatically carried out using Blast2GO with an E-value cut-off ≤10^− 6^ [34]. The tool called WEGO (Web Gene Ontology Annotation Plot) was used for plotting GO annotation GO [45]. The completeness of the gene prediction was assessed using BUSCO v3 [46] software package and pezizomycotina_odb9 gene set.

### Phylogenomic reconstruction

To reconstruct a phylogenetic hypothesis for nine Phyllosticta species (*P. capitalensis* strains: CBS128856, Gm33, LGMF01; *P. citricarpa* strains: CBS141350, Gc12, LGMF06, *P. citribraziliensis* strain CBS100098, *P. citrichinaensis* strain CBS130529), in total, we used 3.185 single copy-ortholog genes identified with OrthoFinder [47]. Proteins sequences were aligned using MAFFT v7.271 [48] and trimmed using TrimAL v1.4 [49]. The Maximum Likelihood (ML) phylogenetic analysis was performed using IQtree v1.6.5 [50], with support was assessed via 1000 bootstrap replicates using the JTT + F + R4 model. The model of nucleotide sequence evolution was inferred using software ModelFinder [51] with both the Akaike information criterion (AIC) [52]. The root of ML tree was inferred the midpoint rooting method [53].

### Gene set enrichment analysis

To realize a functional enrichment analysis of *P. citricarpa* and *P. capitalensis,* protein sequences was submitted to a search of orthologous groups based on reciprocal best hits between each fungus and the yeast reference organism *Saccharomyces cerevisiae*. The search of orthologous groups was performed using the Orthofinder v2.2.7 software. Subsequently, we have selected groups present only in the genomes of *P. capitalensis*/*Saccharomyces* and *P. citricarpa*/*Saccharomyces*. The set of genes retrieved was submitted for GO enrichment analysis in the PANTHER classification system [54] and Saccharomyces Genome database (SGD) [55].

### Expansion and retraction of gene families

We used the maximum-likelihood approach implemented in BadiRate v1.35 [56] to estimate the gene birth, death, and innovation rates in the *Phyllosticta* gene families during the evolution. In brief, we first inferred orthologous groups based on reciprocal best hits within and between gene families of nine *Phyllosticta* genomes using the Orthofinder v2.2.7 software [47]. Single-copy orthologists and lineage-specific gene families were extracted using using in-house Perl script. The ML tree previously inferred from the 3.185 single copy orthologs and orthologous gene groups were utilizeded by the program as input and with the following parameters: -anc 1 -bmodel FR -model BDI -ep ML. For each orthologous group, gene gain and loss events were counted from the number of members at internal nodes inferred by maximum likelihood under the BDI stochastic model [57], assuming that each branch has its own specific turnover rates.

### Comparative genomics

Comparative genomics analysis between *P. citricarpa* and *P. capitalensis* were conducted using only exclusive sequences for each species. These sequences were obtained after different rounds of BLAST [29]. Initially, a blastp analysis was carried out with unique coding sequences extracted from the genome of each organism using Augustus against the proteins from the genomes of the other species. The results were filtered in order to just get hits presenting E values >1e-50. These subsets of coding sequences were then evaluated reciprocally against proteomes available at the JGI (*Phyllosticta citricarp*a, CBS 127454 v 1.0; *Phyllosticata capitalensis*, CBS 128856, v 1.0) using blastp. Once again an E value filter (1e-50) was used and only the sequences showing results below the cutoff were kept for further analyses, being considered as the exclusive sets for each species.

### PHI-base analyzes of the exclusive genes of *P. citricarpa* and *P. capitalensis*

The *P. citricarpa* and *P. capitalensis*, exclusive gene sequences for each, were used to investigate whether they had been verified to be pathogenic genes using annotations from the PHI-base using blastp (e-value < 0.001) [58].

### Analysis of carbohydrate-active (CAZy) enzymes in *P. citricarpa* and *P. capitalensis*

The CAZymes are involved in plant cell wall degradation. For this reason, the genes of both fungi, pathogenic and endophytic, were subjected to CAZy annotation using dbCAN [59], which is based on the CAZy (Carbohydrate-Active Enzyme) database classification [39].

### Secretome

To verify which proteins are secreted the following pipeline was used: the secreted proteins were identified using SignalP 3.0 [60], which detects the presence and location of signal peptide cleavage sites in proteins. In addition, we also used SecretomeP 1.0f [61] to predict protein secretion by nonclassical pathways. Afterwards, TMHMM [62] was used to identify the sequences that have transmembrane domains that were excluded from further analyses. Therefore, the sequences presenting signal peptide and no transmembrane domains were selected. These sequences were then submitted to Cello 2.5 [63] analysis to predict cellular localization of the sequences.

## Additional files

Additional file 1: Sequences of *P. citricarpa* proteins. The proteins obtained after de novo assembly of *P. citricarpa* genome were used for coding sequences prediction using *Botrytis cinerea* as reference in Augustus, when 15,206 proteins were obtained. (TXT 5151 kb)

Additional file 2: Sequences of *P. capitalensis* transcripts. The proteins obtained after de novo assembly of *P. capitalensis* were used for proteins prediction using *Botrytis cinerea* as reference in Augustus, when 14,797coding sequences were obtained. (TXT 5895 kb)

Additional file 3: BUSCO assessment of genomes from Phyllosticta. The genome-level benchmarking value of *P. capitalensis* LGMF01 was C: 65.2% (containing S: 65.1%, D: 0.1%, F: 23.9%, M: 10.9%, n: 3156) and *P. citricarpa* LGMF06 was C:46.9% (containing: S: 46.9%, D: 0.0%, F: 33.7%, M: 19.4%, n: 3.156). The corresponding protein-level benchmarking value was C: 57.5% (including S: 57.4%, D: 0.1%, F: 26.5%, M: 16.0%, n: 3.156) and C: 40.0% (including S: 40.0%, D: 0.0%, F: 35.7%, M: 24.3%, n:3156. Light-blue: complete (C) and single-copy (S) genes; dark-blue: complete and duplicated genes (D); yellow: fragmented genes (F); red: missing genes (M) and n: total BUSCO groups for searching. *P. citriasiana* was used with genome control. (PDF 592 kb)

Additional file 4: Comparative analysis of gene gain and loss rates. Comparative analysis of gene gain and loss rates is represented in relation to different branches from the pathogenic and endophytic Phyllosticta species. (XLSX 41 kb)

Additional file 5: Unique sequences of proteins of *P. citricarpa*. Unique proteins identified after comparison of the *P. citricarpa* and *P. capitalensis* sequences by Blastp. (FA 654 kb)

Additional file 6: Unique proteins of genes of *P. capitalensis*. Unique proteins identified after comparison of the *P. capitalensis* and *P. citricarpa* sequences by Blastp. (FA 992 kb)

Additional file 7: Pathogenicity proteins of *P. citricarpa*. List of *P. citricarpa* exclusive proteins displaying similarity with subjects in the pathogen-host interaction gene database (PHI-base). (XLSX 18 kb)

Additional file 8: Pathogenicity proteins of *P. capitalensis*. List of *P. capitalensis* exclusive proteins displaying similarity with subjects in the pathogen-host interaction gene database (PHI-base). (XLSX 22 kb)

Additional file 9: Comparison of the pathogenicity proteins of *P. citricarpa* and *P. capitalensis*. List of exclusive and common pathogenicity proteins identified after comparison of *P. citricarpa* and *P. capitalensis*. (XLSX 18 kb)

Additional file 10: Secreted CAZymes by *P. citricarpa* and *P. capitalensis*. List of secreted CAZymes identified among the exclusive proteins of each species. (XLSX 9 kb)

## Data Availability

This Whole Genome Shotgun project has been deposited under Bioproject accession PRJNA486917.

## Abbreviations

AA: Auxillary activities
ABC: ATP-binding cassette
acetyl-CoA: Acetyl-coenzyme A
CAZymes: Carbohydrate-active enzymes
CBMs: Carbohydrate-binding modules
CBS: Citrus black spot
CE: Carbohydrate esterases
CFN: Citrus medium fabris-nishimura
GHs: Glycoside hydrolases
GO: Gene ontology
GTs: Glycosyl transferases
MFS: The major facilitator superfamily
ORFs: Open read frames
PHI-base: Pathogen-host interaction gene database
ROS: Reactive oxygen species
WEGO: Web gene ontology annotation plot
