# Supplementary figures and images for: Comparative genome analysis of Phyllosticta citricarpa and Phyllosticta capitalensis, two fungi species that share the same host
Source: BMC Genomics. 2019 Jul 5;20:554. doi: 10.1186/s12864-019-5911-y (PMC6612112; doi:10.1186/s12864-019-5911-y)

***Phyllosticta capitalensis* LGMF01**

**C: 1.817 (57.5%) [S: 1.813 (57.4%), D: 4 (0.1%)], F: 837 (26.5%), M: 502 (16%), N: 3.156**

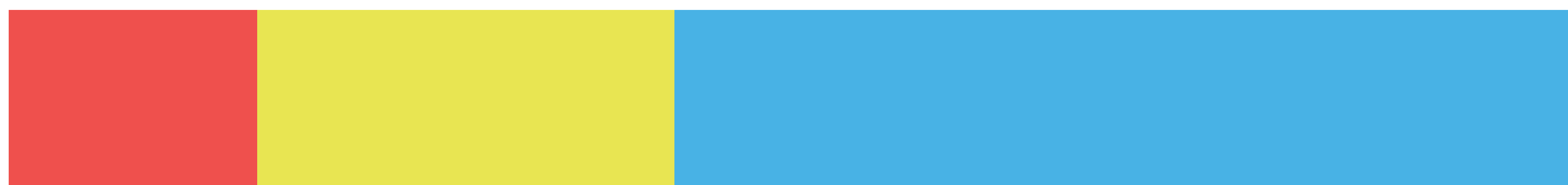

***Phyllosticta citricarpa* LGMF06**

**C: 1.263 (40%) [S: 1.263 (40%), D: 0], F: 1.128 (35.7%), M: 765 (24.3%), N: 3.156**

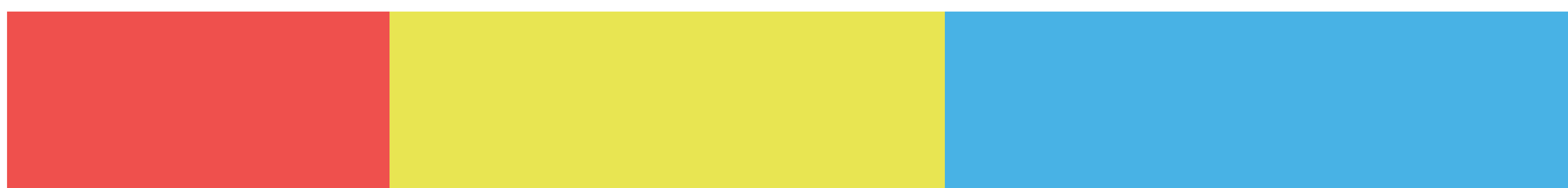

0 20 40 60 80 100  
% BUSCOs

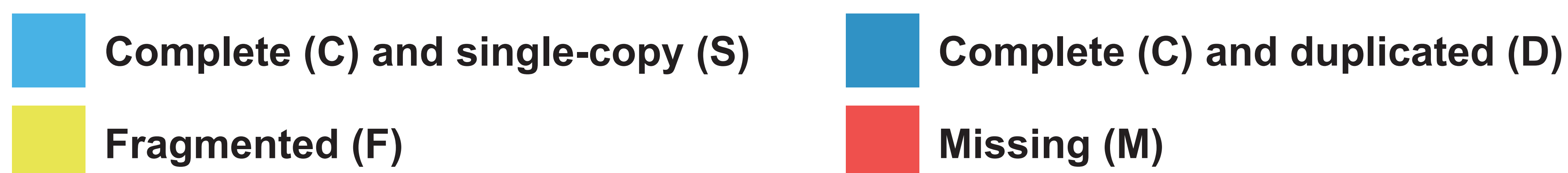

Supplement: Supplementary file 3 — BUSCO assessment of genomes from Phyllosticta. The genome-level benchmarking value of P. capitalensis LGMF01 was C: 65.2% (containing S: 65.1%, D: 0.1%, F: 23.9%, M: 10.9%, n: 3156) and P. citricarpa LGMF06 was C:46.9% (containing: S: 46.9%, D: 0.0%, F: 33.7%, M: 19.4%, n: 3.156). The corresponding protein-level benchmarking value was C: 57.5% (including S: 57.4%, D: 0.1%, F: 26.5%, M: 16.0%, n: 3.156) and C: 40.0% (including S: 40.0%, D: 0.0%, F: 35.7%, M: 24.3%, n:3156. Light-blue: complete (C) and single-copy (S) genes; dark-blue: complete and duplicated genes (D); yellow: fragmented genes (F); red: missing genes (M) and n: total BUSCO groups for searching. P. citriasiana was used with genome control. (PDF 592 kb) [file 12864_2019_5911_MOESM3_ESM.pdf]
